# Plasma and Platelet Brain-Derived Neurotrophic Factor (BDNF) Levels in Bipolar Disorder Patients with Post-Traumatic Stress Disorder (PTSD) or in a Major Depressive Episode Compared to Healthy Controls

**DOI:** 10.3390/ijms25063529

**Published:** 2024-03-20

**Authors:** Valerio Dell’Oste, Lionella Palego, Laura Betti, Sara Fantasia, Davide Gravina, Andrea Bordacchini, Virginia Pedrinelli, Gino Giannaccini, Claudia Carmassi

**Affiliations:** 1 Department of Clinical and Experimental Medicine, University of Pisa, 56126 Pisa, Italy; lionella.palego@unipi.it (L.P.); dr.fantasiasara@gmail.com (S.F.); davide.gravina@hotmail.it (D.G.); andreabordacchini@gmail.com (A.B.); virginiapedrinelli@gmail.com (V.P.); claudia.carmassi@unipi.it (C.C.); 2 Department of Biotechnology, Chemistry and Pharmacy, University of Siena, 53100 Siena, Italy; 3 UFCSMA Zona Valdinievole, Azienda USL Toscana Centro, 51016 Montecatini Terme, Italy; 4 Department of Pharmacy, Section of Biochemistry, University of Pisa, 56126 Pisa, Italy; laura.betti@unipi.it (L.B.); gino.giannaccini@unipi.it (G.G.); 5 UFSMA Zona Apuana, Azienda USL Toscana Nord Ovest, 54100 Massa, Italy

**Keywords:** bipolar disorder (BD), post-traumatic stress disorder (PTSD), major depressive episode, plasmatic brain-derived neurotrophic factor, intraplatelet brain-derived neurotrophic factor

## Abstract

Post-traumatic stress disorder (PTSD) is a highly disabling mental disorder arising after traumatism exposure, often revealing critical and complex courses when comorbidity with bipolar disorder (BD) occurs. To search for PTSD or depression biomarkers that would help clinicians define BD presentations, this study aimed at preliminarily evaluating circulating brain-derived-neurotrophic factor (BDNF) levels in BD subjects with PTSD or experiencing a major depressive episode versus controls. Two bloodstream BDNF components were specifically investigated, the storage (intraplatelet) and the released (plasma) ones, both as adaptogenic/repair signals during neuroendocrine stress response dynamics. Bipolar patients with PTSD (*n* = 20) or in a major depressive episode (*n* = 20) were rigorously recruited together with unrelated healthy controls (*n* = 24) and subsequently examined by psychiatric questionnaires and blood samplings. Platelet-poor plasma (PPP) and intraplatelet (PLT) BDNF were measured by ELISA assays. The results showed markedly higher intraplatelet vs. plasma BDNF, confirming platelets’ role in neurotrophin transport/storage. No between-group PPP-BDNF difference was reported, whereas PLT-BDNF was significantly reduced in depressed BD patients. PLT-BDNF negatively correlated with mood scores but not with PTSD items like PPP-BDNF, which instead displayed opposite correlation trends with depression and manic severity. Present findings highlight PLT-BDNF as more reliable at detecting depression than PTSD in BD, encouraging further study into BDNF variability contextually with immune-inflammatory parameters in wider cohorts of differentially symptomatic bipolar patients.

## 1. Introduction

Post-traumatic stress disorder (PTSD) is a severe psychiatric disorder typically occurring after exposure to a traumatic event, causing chronic psychological suffering and leading to an often chronic and invalid course [1,2]. According to the current DSM-5-TR classification [3], PTSD is characterized by four clusters of symptoms, namely intrusion (criterion B), avoidance (criterion C), negative alterations in cognitions and mood (criterion D) and alterations in arousal and reactivity (criterion E). Some neurobiological mechanisms might play a significant role in the development of PTSD after traumatic experiences in these subjects. The biological heterogeneity and variable symptom presentation of PTSD suggest the need for biomarkers that reflect multiple biological measures.

The sequential responses to recurrent and chronic stress by the hypothalamic–pituitary–adrenal (HPA) axis and the autonomic nervous system are considered to play a significant role in the onset and progression of PTSD [4]. Decreased activity of the HPA axis and parasympathetic nervous system, along with increased activity of the sympathetic nervous system, have been observed in PTSD, which may lead to impaired immune system reactivity, defined by the unbalanced release of proinflammatory cytokines [5,6]. Such variations in the immune system may modify the synaptic structure and function in brain regions through changes in serotonin metabolism, direct and potentially neurotoxic effects of cytokines and oxidative stress, as well as the decreased action of some specific neurotrophins such as brain-derived neurotrophic factor (BDNF) [5]. Neurotrophins represent a family of regulatory factors able to mediate neuronal differentiation and survival, as well as modulate signal transmission and synapse plasticity [7,8]. In particular, BDNF is the most abundant neurotrophin in the brain [9] and plays an important role in promoting the survival and differentiation of different neuronal populations in the prenatal period [10]. This neurotrophin has been detected also in the peripheral nervous system, where it modulates the proliferation, differentiation and activity of specific neuronal populations [11,12]. The BDNF promotes both long- and short-term effects in the adult nervous system [13]: on the one hand, it acts as a powerful excitatory neurotransmitter by determining the rapid depolarization of the postsynaptic membranes, even at low concentrations [14,15], while on the other, it determines long-term modifications of synaptic plasticity and production of neurotransmitters and neuropeptides [16,17,18]. Thus, BDNF is believed to have long-lasting effects on mental processes such as memory, learning and adaptive behavior [17,18]. The BDNF also promotes acute effects on synaptic plasticity and neurotransmitter release, facilitating the release of glutamate, γ-aminobutyric acid (GABA), dopamine and serotonin [19]. In support of this, several pieces of evidence obtained in rodent models have shown the relevance of BDNF in stress resilience and brain fear circuitries [20,21,22].

Over the years, increasing evidence demonstrated the role of BDNF in the pathophysiology of stress-related disorders as well as in the context of the complex relationships between stressful life events, acute or chronic, and the onset or exacerbation of mood disorders [23,24]. According to the neurotrophic hypothesis of depression, the latter may be linked to a deficit of neurotrophic factors, which can be resolved with antidepressant therapy [25,26]. Also, in PTSD, the downregulation of BDNF, accompanied by consequent changes in neuronal plasticity occurring in response to stress, is thought to contribute to the severe behavioral alterations and clinical manifestations observed in patients [27].

Parallel to the molecular studies conducted to search for appropriate peripheral biomarkers of mood disorders and depression, the above findings prompted the investigation of PTSD correlates as well, through noninvasive procedures, as a reflection of the altered neuroendocrine responses in this mental condition. Among the various parameters examined, peripheral BDNF has acquired an ever-growing interest in clinical psychiatry [28]. Both the BDNF mRNA and protein have been revealed in the bloodstream [29,30,31], where this neurotrophin has been found to be about ten times more concentrated in serum than plasma [32]. A BDNF component has been measured in platelets too [29,33]: platelets can capture the neurotrophins from plasma through a mechanism not yet known so that 90% or more of blood BDNF is stored and transported inside them [29,34].

Plasma BDNF can originate from the endothelium and smooth myocytes, as well as activated macrophages and lymphocytes, all BDNF-producing tissues/cells. However, their contribution to the plasma concentration of BDNF is considered marginal under basal conditions compared to the amount released by platelets [32,34]. The role of peripheral BDNF has been linked to the activation of the neuroendocrine CNS stress response cascade, as well as to the immune system response and inflammation, exerting a presumable influence upon the regulation of mood and related behaviors [35,36]. At the same time, the specific function of intraplatelet BDNF levels has not yet been fully elucidated; probably, it acts under specific tissue trauma, nerve injury and hemorrhages. Indeed, activated platelets release BDNF from their granules, probably playing reparative actions during inflammation and smooth muscle cell proliferation. The ability of BDNF to cross the blood–brain barrier (BBB) has been described [37], and a study by Karege and coauthors in rats [38] has shown that brain and serum levels of BDNF undergo parallel changes during growth and development processes. So, despite the conflicting evidence regarding the BDNF passage across the BBB [37,39,40], the possibility that this neurotrophin can cross it in both directions [37] suggests that the cerebral BDNF concentrations are in equilibrium rather with its amount in the plasma than in the serum, where the neurotrophin levels are closely related to platelet release [41]. Indeed, serum BDNF levels also include the BDNF platelet component released during the activation of the coagulation process [29,42,43].

The difference between the serum and plasma BDNF levels has been thought to indicate the amount of BDNF stored in circulating platelets, but BDNF is not fully released during platelet activation: it has been indeed shown that platelets either have a nonreleasable pool of BDNF and/or that the released BDNF is sequestered by binding to a transporter or receptor on the platelet surface [29,42,43]. It is noteworthy that, more recently, some authors have reported that human megakaryocytes can instead produce BDNF as transcripts and translated proteins, similarly to neurons, suggesting that platelets can have their own endogenous neurotrophin reserve [44]. It is, therefore, conceivable to think that there is a complex equilibrium among all the diverse intra- and extraplatelet BDNF components [42,43,45,46]. These findings support the importance of measuring BDNF within platelets since they seem not only to be a “passive” transport source of BDNF in the bloodstream but they are also supposed to establish active in- and outflows of the neurotrophin to ensure its proper compartmentalization and distribution in tissues under different activation states [42].

Studies conducted on circulating BDNF, mainly the plasma and/or serum pools, as a correlate of stress and PTSD have produced quite conflicting results until now, needing deeper investigation. Mitoma and coauthors [47] found a statistically significant negative correlation between stress and serum concentrations of BDNF. Other authors have also reported lower BDNF levels in the plasma or serum of PTSD patients [48,49,50,51,52]. Several neurobiological models on reduced BDNF levels and function in PTSD have been proposed, including trauma-induced epigenetic regulation and current stress [22,27]. Anyway, other surveys obtained the opposite, finding higher plasma or serum BDNF levels in these patients [53,54,55,56], which were also in relation to specific traumatisms, symptoms or trauma exposure frequency. Also, a recent systematic review of twenty studies on serum or plasma BDNF levels showed rather increased levels in PTSD patients versus healthy controls [57].

There is also an additional degree of complexity for this kind of investigation, essentially related to the fact that clinical and epidemiological data have often noted the strong association between PTSD and other psychiatric disorders, including mood disorders [58]. This has led to considering comorbidity in PTSD to be the rule rather than the exception [58,59,60,61], determining a significantly poorer outcome, higher symptom burden, lower quality of life, higher percentage of further concurrent psychiatric disorders and higher suicide risk [62,63,64]. Additionally, a huge number of clinical and epidemiological studies emphasized different psychopathological and neurobiological hypotheses underlying PTSD and BD, proposing peculiar pathways for these disorders and explaining, at least in part, the conflicting results observed until now. While scant data are currently available in the literature about the potential biomarkers of PTSD, even less have been obtained on the possible comparisons of PTSD and depressive state biomarkers in subjects affected by BD. The aim of the present study was therefore to evaluate two different BDNF pools in the bloodstream, plasma and platelets, in a group of BD subjects in the euthymic phase showing PTSD symptoms (PTSD group) compared with a group of BD subjects experiencing a major depressive episode (DEP group) and a healthy control group (CTL group).

The plasma BDNF component was taken as the extracellular pool of the circulating neurotrophin, or the cell/tissue-released one, whereas the PLT-BDNF content was considered the bloodstream storage/tissue distribution counterpart in the periphery by investigating both of them as players of stress-related neuroendocrine networks [5,35,36,65].

## 2. Results

### 2.1. Subjects Characteristics

Our sample included 20 BD subjects in the euthymic phase with PTSD (PTSD group), 20 BD subjects experiencing a major depressive episode (DEP group) and 24 healthy controls (CTL group). The mean age in the total sample was 41.38 ± 14.98 years, 42.10 ± 15.11 years for the PTSD group, 50.35 ± 16.41 years for the DEP group and 33.29 ± 8.05 years for the CTL group. The total sample included 42 (65.6%) females and 22 (34.4%) males. The PTSD group included 15 (75.0%) females and 5 (25.0%) males, the DEP group included 12 (60.0%) females and 8 (40.0%) males and the CTL group included 15 (62.5%) females and 9 (37.5%) males, a gender distribution that did not differ significantly between the three groups.

Among the patients, 11 subjects (27.5%) were at the point of first contact with a psychiatric service, while 29 (72.5%) were at a follow-up visit; specifically, 30 (75.0%) were inpatients while 10 (25.0%) were outpatients. All patients had a lifetime BD diagnosis: in particular, in the PTSD group, five subjects (25.0%) had a BD type I diagnosis and 15 (75.0%) had a BD type II diagnosis, while in the DEP group, eight (40.0%) and twelve (60.0%) had BD type I and type II diagnosis, respectively. All recruited patients were undergoing pharmacological treatment and were stable with respect to drugs and dosages for at least one month, according to the established inclusion criteria. Thirty-four (85.0%) patients were receiving antidepressant treatment, 33 (82.5%) were receiving a mood stabilizer, 30 (75.0%) were receiving an antipsychotic and 24 (60.0%) were receiving a benzodiazepine drug. Further, the mean value of a year of illness in patients was 15.18 ± 12.75 years.

In the PTSD group, 13 (65.0%) subjects reported having been exposed to actual or threatened death, 4 (20.0%) to serious physical injury and 3 (15.0%) to sexual violence. Particularly, 13 (65.0%) reported to have directly experienced a traumatic event, while 7 (35.0%) witnessed, in person, the event as it occurred to others. The mean value of years after the traumatic event in patients with PTSD was 7.37 ± 12.52 years, with 12 subjects (60%) reporting a traumatic event that occurred in the last year. Finally, seven (35.0%) reported a traumatic event related to the COVID-19 pandemic. In the DEP group, although none reported ongoing post-traumatic symptoms or a “major” traumatic event, the following lifetime “minor” potentially traumatic events were reported when fulfilling the IES-R symptomatologic domains: interpersonal violence (7, 35.0%), the breakup of a romantic relationship (4, 20.0%), a grief-related event (3, 15.0%), a chronic illness (3, 15.0%) and being bullied (3, 15.0%). We also performed a comparison of the scores reported on the psychometric scales among groups (Table 1).

PTSD and DEP groups reported higher scores than the CTL group on all domains for each instrument. The DEP group presented higher HAM-D total scores than the PTSD group (18.40 ± 3.55 vs. 8.40 ± 2.41, *p* < 0.001), while the PTSD group exhibited higher IES-R total (50.42 ± 11.18 vs. 11.55 ± 6.99, *p* < 0.001) and Intrusion (2.39 ± 0.90 vs. 0.51 ± 0.36, *p* < 0.001), Avoidance (2.14 ± 0.69 vs. 0.53 ± 0.40, *p* < 0.001) and Hyperarousal (2.37 ± 0.75 vs. 0.54 ± 0.37, *p* < 0.001) IES-R domain scores. No differences in the YMRS total score emerged in the two patient groups (*p* = 0.736).

### 2.2. BDNF Comparisons and Correlations

**Figure 1:**
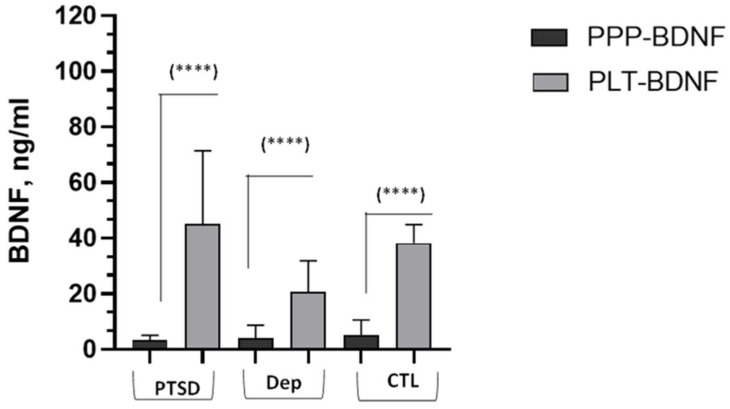
Comparison of PPP-BDNF vs. PLT-BDNF levels (ng/mL) within each group. Bar histograms represent the BDNF mean and SD of n = 20 BD patients with PTSD (PTSD), n = 20 BD patients in a major depression episode (DEP) and n = 24 healthy controls (CTL). (****): Wilcoxon test, *p* < 0.0001, significantly higher amounts of PLT-BDNF than PPP-BDNF.

Concerning the BDNF results, Figure 1 depicts the neurotrophin values obtained in the PPP and in the platelet lysates, both reported in ng/mL. The picture shows that platelet contents were markedly higher inside platelets than in the plasmatic fraction in subjects from all three groups under investigation. The PLT-BDNF levels, reported in the figure as ng/mL but applying a proportional correction for average total protein amounts in PRP, were 45.16 ± 26.32 ng/mL in the PTSD group and 20.53 ± 11.32 ng/mL and 38.17 ± 6.69 ng/mL in the depressed and control subjects, respectively. The PPP-BDNF values were those reported in Table 1. The within-group pairwise Wilcoxon comparison of PLT-BDNF vs. PPP-BDNF amounts (ng/mL, see the Materials and Methods section), reported markedly greater BDNF levels inside platelets than in PPP (Figure 1).

**Figure 2:**
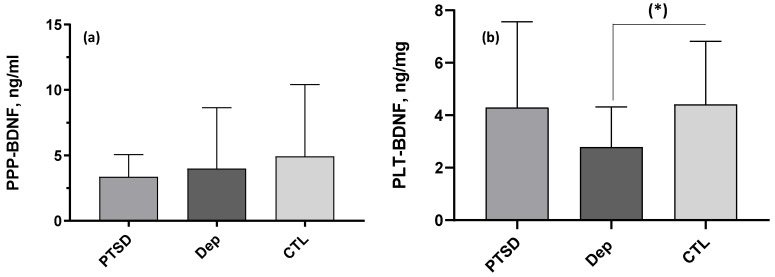
Comparison of (**a**) PPP- and (**b**) PLT-BDNF levels among groups. The bar histograms represent the mean and SD of BDNF levels measured in BD patients with PTSD (n = 20, PTDS), in BD patients in a major depressive episode (n = 20, DEP) and in healthy controls (n = 24, CTL). (**b**) (*): significant Kruskal–Wallis analysis, *p* < 0.05.

In addition, the nonparametric Kruskal–Wallis analysis of variance showed that PLT-BDNF levels (normalized for mg proteins) were significantly lower in the DEP group when compared with the CTL one. The Kruskal–Wallis analyses for (a) platelet-poor plasma PPP-BDNF (ng/mL) and (b) intraplatelet PLT-BDNF (ng/mg proteins) in the three groups under investigation showed a significant post hoc Dunn test, with reduced PLT-BDNF in depressed patients (DEP) vs. controls (CTL), (*) *p* < 0.05. No differences emerged in the PPP-BDNF levels among the three groups. Details are reported in Table 1 and Figure 2.

**Figure 3:**
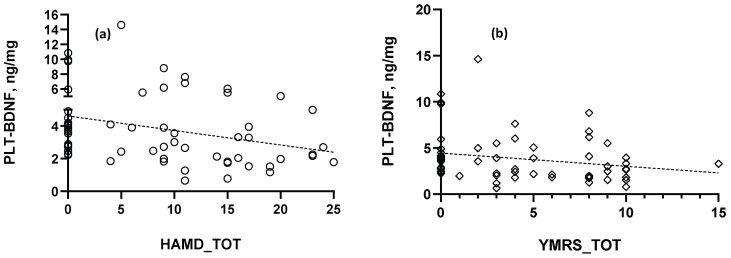
Significant Spearman correlations obtained between PLT-BDNF and (**a**) depression or (**b**) mania rating scores. Dotted lines in each graph represent the best-fit correlation trajectories obtained after a linear regression analysis of data.

No significant Spearman correlation occurred between PPP-BDNF and PLT-BDNF levels, whereas PLT-BDNF concentrations were found negatively correlated with both the HAM-D and the YMRS total scores: (a) R_S_ = −0.369, (**) *p* < 0.01; (b) R_S_ = −0.31, (*) *p* < 0.05, respectively (Figure 3). Conversely, no significant correlation was reported for PPP-BDNF related with HAM-D depression scores, displaying a p value near the statistical significance level (R_S_ = −0.21, *p* = 0.09). 

Of note, a tendency toward a positive correlation was reported between PPP-BDNF levels and YMRS total results (R_S_ = 0.22, *p* = 0.08). Both BDNF components were not significantly correlated with scores of the IES-R psychometric scale (PLT-BDNF, R_S_ = −0.1; PPP-BDNF, R_S_ = −0.15, *p* > 0.05), specific for defining PTSD clinical presentation.

## 3. Discussion

To our knowledge, the present study is the first that evaluated and compared the amount of two circulating components of the neurotrophin BDNF, the platelet-poor plasmatic (PPP-BDNF) and the intraplatelet (PLT-BDNF) pools, in a group of patients with a lifetime diagnosis of bipolar disorder, distinguished with respect to the presence of current post-traumatic symptoms or depressive episodes, and a group of healthy controls without somatic or mental diseases. Essentially, these two BDNF circulating parameters were evaluated as presumed biomarkers of the activity of the CNS periphery communication networks involving the neurotrophin system in these groups of enrolled subjects [35,36,41,42,65,66]. First, results showed that the two BDNF parameters were not significantly correlated to each other, in support of their distinct origin [29,43,67]. The contents of intraplatelet BDNF were found strongly and in significantly greater concentrations than in PPP within the same subject group and in all groups, confirming the role of platelets in the transport and storage of BDNF in the bloodstream [29,32], as well as that they were active in this function in all recruited subjects. Moreover, BDNF levels were higher, ranging from 5 to 100 times on average, in platelets than PPP, which is in agreement with other authors [42]. After adjustment for protein contents, the PLT-BDNF levels, as ng/mg proteins, were found significantly reduced in depressed patients vs. healthy controls, displaying significant negative correlations with mood psychometric results or the HAM-D and YMRS scores. As concerns the extracellular and plasmatic pool appraised herein, for the PPP-BDNF, we reported different results from previous studies showing a significant reduction in plasma BDNF in major depression, although our group of depressed patients displayed PPP-BDNF mean values and a mean rank lower than PTSD patients and healthy controls. Some previous data showed, in fact, that depressed patients have lower BDNF plasma levels than controls, suggesting that plasma BDNF may parallel central BDNF and may be associated with clinical features of major depressive disorder [68,69,70]. A meta-analysis has also shown that BDNF is strongly reduced in severe depression while increasing during antidepressant treatment, even in the absence of clinical remission [71]. Other studies reported low BDNF levels to be related to more severe melancholic characteristics, psychomotor retardation or slowdown and insomnia [72,73]. Furthermore, a negative correlation between BDNF (plasma or serum BDNF) and the number of depressive episodes with melancholic features was found [74]. These results support the hypothesis that lower plasma BDNF reflects the reduction in central BDNF, considering that decreased BDNF levels have been found in the brains of suicide subjects, depressed patients and stressed animals [75,76,77]. Environmental stressors like psychological stress, chronic foot shocks and chronic social defeats have been also found to decrease BDNF levels in the hippocampus [36,77]. Additionally, a decrease in circulating BDNF has been associated with mood episodes in BD, including mixed-state episodes, though with slightly less reduced values than in depressed patients [78].

Our result showing nonsignificant PPP-BDNF levels in patients vs. controls still requires attention. The BDNF values reported in the investigations conducted until now have been quite variable between the different studies [34,41,65,67,78,79,80,81,82,83,84]. The influence of many parameters can affect plasma BDNF measures [67]: different procedures in blood withdrawal, plasma separation and handling, centrifugation, temperature and plasma acidification may all impact final plasma BDNF results [42,65,85,86]. For instance, plasma acidification, used by some of the above-mentioned authors, enables the measure of greater levels of BDNF, including the BDNF-bound forms [87]. Higher levels of BDNF have been also reported in patients under drug treatment with antidepressants versus the drug-naïve ones [42]. The specificity of the capture antibodies used in the ELISA assay kit and the timing of blood handling after withdrawal have been questioned and indicated as possible variability factors [88]. In the present study, most of these aspects were controlled and have been carefully considered, preventing our results from being affected by these critical points. It is, however, always possible that BDNF amounts in PPP, obtained by two different centrifugation steps, are not fully identical to the plasmatic ones [42,67], and they could reflect alterations in inflammatory components or pathways, as observed in different cohorts of depressed patients [42,80,84]. Substantially, plasmatic BDNF may rather be a state marker related to the presence of peculiar proinflammatory paths activated in different clinical presentations, including production and release by the endothelium, smooth musculature and activated lymphomonocytes [42], as well as the intrinsic variability in extracellular BDNF as a modulator of the allostatic response to stress and its dynamics [42]. In this framework, it should be also mentioned that even increased serum or plasma BDNF levels have been obtained in mood disorder patients under peculiar states such as a high cytokine IL-1β production, in bipolar disorder-II vs. subthreshold bipolar patients or in pain disturbances and in adaptive disorders [42,65,78,80,89,90,91,92]. Also, in the present study, PPP-BDNF showed a trend towards a negative correlation with HAM-D depression scores, following the PLT-BDNF trajectory, but also an opposite tendency to a positive correlation with the manic items, suggesting that this parameter can be under the influence of other pathways, potentially altered in counter-polar mood states [84,92].

These considerations can also be applied to explain our results obtained for PPP-BDNF levels in PTSD: in this case, the few studies conducted on plasma (and/or serum) BDNF reported in the current literature are even more contrasting than those obtained for depressed patients. As suggested by some authors, in these patients, there could be an impaired activity of the sympathetic system and HPA [5], which could peculiarly affect BDNF plasma amounts [93]. Moreover, BDNF has been reported to display a biphasic release into the bloodstream with respect to the initial trauma by increasing in the first acute reactive phase and by decreasing later [94]. The biphasic “shape” of the BDNF peripheral response in relation to high-stress conditions could be the origin of the discrepancies obtained in the investigations conducted until now on plasma BDNF in PTSD since different experimental conditions were adopted with respect to time elapsed from the traumatic event. Our patients experienced a traumatic event within, on average, 7 years of the study, implying a relatively prevalent chronic clinical condition. Our negative results concerning altered BDNF in patients with PTSD symptoms suggest that BDNF seems to not be linked to PTSD symptoms or that, as detailed before, it is a parameter under the influence of stress-dependent networks involving specific PTSD features. The recent meta-analysis of Mojtabavi and coauthors [57], reporting an increased plasma BDNF in PTSD patients, points out some of these aspects while encouraging the pursuit of investigations on BDNF and PTSD considering a wider cohort of patients to permit their distinction with respect to the chronological distance from the traumatic event and the kind of experienced traumatism.

The PLT-BDNF amounts were found instead significantly reduced in depressed patients versus controls. Very few studies have investigated BDNF contents inside platelets of psychiatric patients. In addition, both basic and clinical studies from the literature were conducted, adopting different procedures of platelet lysis and homogenization [29,45,80,84,95,96]. Most authors normalized BDNF values obtained in platelet lysates with respect to platelet counts measured by flow cytometry in the PRP. In the present study, we chose to normalize them for the total protein content of platelet lysates, which is quite proportional to the platelet counts, while reflecting the final intraplatelet fraction yields. To indicatively compare PLT-BDNF amounts in ng/mg proteins with those reported in the ng/plt numbers, we have related intraplatelet BDNF values, directly obtained as ng/mL from the calibration curve and sample dilution adjustment, to a mean estimated platelet number in PRP. In this way, our PLT-BDNF levels were comparable to those obtained by Fujimura and coauthors [29], being slightly higher than those reported by Lee and Kim [96] and Serra-Millàs and coauthors [80]. Despite the different procedures employed to obtain platelet fractions and to normalize PRP BDNF values, the statistical results presented by these authors [80,96] matched ours: PLT-BDNF was always found significantly reduced in depressed patients. Moreover, the fact that the intraplatelet neurotrophin was found negatively correlated with both the depression HAM-D and mania YMRS scores suggests that this component is more reliable and less variable than the extracellular one in the bloodstream, being, putatively, a more valuable peripheral biomarker of mood disorders. Platelets are the main BDNF storage secreted from all other tissues [32,33] and, when activated, release numerous inflammatory mediators, including BDNF [97]. Up to 40–70% of their BDNF total content could be released when platelets are activated [29]. The PLT-BDNF can be more stable than PPP-BDNF maybe because platelets either have a non-releasable pool of BDNF or megakaryocytes endogenously produce BDNF [44], and the released BDNF is sequestered by binding to a transporter or receptor on the platelet surface [29]. The PLT-BDNF counterpart appears rather under the influence of lifetime mood-related parameters. In a previous work, we reported that PLT-BDNF was reduced in patients with a major depressive disorder with respect to those with a diagnosis of BD-I, being also negatively correlated with HAM-D scores [84], thus entailing that both lifetime and current depression seem to exert a kind of “hierarchical” impact on this parameter. This would also explain the lack of variance in PLT-BDNF in PTSD symptomatic bipolar patients with respect to controls, even if this parameter displayed mean and mean rank values higher than depressed patients but lower than controls, without attaining the statistical significance in both cases. Also, no significant correlation was observed with respect to the IES post-traumatic scale, additionally implying that PLT-BDNF is a better indicator of mood symptoms and depressive conditions.

The present survey presents some limitations that should be mentioned. First, it was conducted following a cross-sectional experimental design in small subgroups of subjects, even if the selection criteria were rigorous, allowing us to recruit patients from a naturalistic perspective, thus reflecting the clinical reality. For the same reasons, all patients were assessed under a pharmacological intervention, a condition that, although stable at the time of the investigation, could have an impact on BDNF results. Other limitations are also due to the incomplete data recording concerning the nutritional state, smoking behavior and physical activity of subjects enrolled in the investigation.

## 4. Materials and Methods

### 4.1. Subjects

Patients with bipolar disorder (BD) were recruited at the Psychiatry Unit of the Santa Chiara University-Hospital, Department of Clinical and Experimental Medicine, (AOUP) Pisa, Italy. Adult out- or inpatients were evaluated, selected and admitted to the study from those presenting in a current euthymic phase with a PTSD diagnosis (PTSD group) as well as those with a current major depression episode diagnosis (DEP group). Voluntary subjects, in good mental and physical condition, were also evaluated and recruited from a pool of healthcare professionals at the hospital as the control group for this survey (healthy controls, CTL group).

Inclusion criteria for patients were as follows: age between 18 and 65 years; history and diagnosis of BD, according to the classification criteria of the DSM-5, in the euthymic phase (only for the PTSD group) or experiencing a major depression episode (only for the DEP group); history and diagnosis of current PTSD according to the classification criteria of the DSM-5 (only for the PTSD group); recruitment at the first visit or follow-up control for outpatients or the first day of hospitalization for inpatients, before any therapeutic intervention or variation in therapies applied at the time of clinical assistance and start of the study, thus comprising only subjects under a stable psychotropic treatment for at least one month; and acceptance of the protocol and signature of informed consent. Exclusion criteria for patients were as follows: age under 18 and over 65 years; history and diagnosis of relevant full-blown neurological or medical diseases; inability to sign an informed consent to the study; alcohol or substance abuse in the last six months; or pregnancy. Exclusion criteria for the CTL group (healthy volunteers) were the same for patients, except that the controls all had to be without a history of or current mental disease according to the DSM-5.

Trained psychiatrists assessed all recruited subjects by means of a structured clinical interview and the administration of suitable psychometric scales. Blood samples were also collected from all participants to appraise the intraplatelet and plasmatic levels of BDNF. The BDNF assays were accomplished at the Laboratory of Biochemistry of the Department of Pharmacy of the University of Pisa. All participants received clear information about the study and had the opportunity to ask questions before providing written informed consent. All data were treated according to Italian and European Privacy laws and rules. The study was conducted in accordance with the Declaration of Helsinki, and all procedures were approved by the local ethical committee (19299/21).

### 4.2. Psychometric Instruments

Sociodemographic and clinical information were registered in a case report form. Participants were investigated by means of the Structured Clinical Interview for DSM-5 disorders (SCID-5) in order to evaluate the presence of a current psychiatric diagnosis [98]; the Hamilton Depression Rating Scale (HAM-D) to assess the actual severity of the depressive symptoms [99]; the Young Mania Rating Scale (YMRS) to appraise the actual severity of the manic symptoms [100]; and the Impact of Event Scale-Revised (IES-R) to assess the actual severity of post-traumatic stress symptoms [101]. Briefly, we describe here all the tests applied for the survey.

The SCID-5 is a diagnostic instrument: the sequence of questions follows the order of the related diagnostic manual (DSM-5), and the different items of each module guide the interviewer through the evaluation of the presence of symptoms that may satisfy the diagnostic criteria [98].

The HAM-D is a 21-item questionnaire that quantitatively assesses the severity of the depressive condition shown by the interviewed subjects and documents all possible changes in patients, taking into account both the extent of symptoms and their frequency. The reported scores define the cut-off severity as follows: score > 25 = severe depression; score between 18 and 24 = moderate depression; score between 8 and 17 = mild depression; and score < 8 = no depression [99].

The YMRS is an 11-item scale that explores key symptoms of mania, generally present throughout the course, from the most modest symptoms to the more serious ones. The YMRS resembles HAM-D in its structure, and the severity assessment is based on what the patient reports about his/her condition in the past 48 h and on the observation of his/her behavior by the doctor during the interview [100].

The IES-R is a 22-item scale measuring three core features of PTSD (re-experiencing of traumatic events, avoidance and hyperarousal): items, coded on a 0–4 scale, are thus divided into three subscales, intrusion, avoidance and hyperarousal, which refer to the last week prior to the assessment. The mean score of the items of each subscale determines the subscale score. The IES-R total score is calculated by adding the score of each item. A score over 32 represents a cut-off for PTSD [101].

### 4.3. BDNF Measurements

#### 4.3.1. Instruments, Chemicals and Reagents

All reagents and chemicals used for the study were of the best quality and purity, in agreement with the gold-standard guidelines of analytical laboratories. All solutions were prepared using ultrapure HPLC gradient-grade milli-Q water (18 MΩ cm resistivity), obtained using a Simplicity Millipore Apparatus equipped with a UV lamp and a 0.2-micron filter to prevent contamination by particles and biological agents. For BDNF dosages, a sandwich ELISA kit, commercially purchased and developed for the preferential determination of the human BDNF mature form (Biosensis, mature BDNF RapidTM, Thebarton, Australia), was employed. This kit allows the colorimetric identification and quantification of BDNF levels in different kinds of biological specimens. It is validated to prevent analytical interferences and its quantitative performances are certified: the antibodies used in the kit mainly react with the mature form of BDNF, avoiding cross-reactions with the precursor pro-BDNF. In particular, this ELISA kit has been updated according to the article of Polacchini and coauthors [88]. The test has a high sensitivity, allowing the detection of BDNF concentrations as low as 7 pg/mL, and a high specificity, with less than 3% of other neurotrophins (such as NT-3, NT-4 and NGF) that can be bound by the first monoclonal anti-BDNF “capture” antibody. A PST-60HL plate thermo-shaker (Biosan, Riga, Latvia) was used for sample incubations during the BDNF ELISA assays when required. Absorbance at 450 nm was appraised using a 96-well plate spectrophotometer (MultiSkan FC ThermoScientific, Thermofisher Scientific, Waltham, MA, USA). The multimodal Enspire reader (Perkin-Elmer, Waltham, MA, USA) was used in pilot experiments to compare the analytical accuracy.

#### 4.3.2. Blood Sampling, Preparation and Storage Procedures

All blood samples were accurately withdrawn, avoiding hemolysis, by the skilled and authorized nursing staff of the Clinical Psychiatric Unit, Department of Clinical and Experimental Medicine, University of Pisa. About 9 mL of peripheral venous blood was collected from all recruited subjects, who had been fasting from the previous evening and at least for 12 h. Blood withdrawals were carried out as part of routine blood tests between 9 and 10 a.m. to avoid circadian rhythm interferences, according to the local ethical committee guidelines for this study. Briefly, blood was gathered in 3 vacutainer tubes containing K_3_EDTA as the anticoagulant. Vacutainer tubes were then immediately transported in thermostatic containers by authorized medical personnel of the Psychiatry Unit to the Biochemistry Laboratory of the Department of Pharmacy, University of Pisa, for sample preparation procedures and proper storage.

Blood was treated to freshly obtain two different biological samples: platelet-poor plasma (PPP) and whole platelets. For PPP and platelet preparation, vacutainer tubes were always centrifuged at the Biochemistry Laboratory within 30 min of withdrawal. All centrifugations were performed at room temperature (RT), 22–25 °C, as previously described [45,84]. The first centrifugation was conducted at low speed, 150–200× *g*, for 15 min, in order to separate the platelet-rich plasma (PRP) from the other cellular elements. Then, the resulting PRP volume was transferred in a Falcon tube (capacity = 15 mL), carefully measured and precisely divided in half into two Falcon tubes and centrifuged at 1500× *g* for 15 min at RT. After this last centrifugation, the PRP yielded two different phases, collected as separate samples: (1) the supernatant containing the K_3_EDTA-PPP and (2) the whole-platelet pellets. The PPP was aliquoted in high-quality, low-binding protein Eppendorf Safe-Lock tubes (Sigma-Aldrich, St. Louis, MO, USA), while whole-platelet pellets were maintained in the Falcon tubes. Specifically, for each patient, a total of about 6–9 aliquots of PPP and two aliquots of platelet pellets were obtained. The PRP volumes, from which PPP and whole-platelet pellets were derived, were registered and saved in the database for the calculation of intraplatelet BDNF levels and protein content analysis. The PPP Eppendorf aliquots and platelet pellets were stored in a −80 °C freezer until the plasmatic PPP-BDNF and the intraplatelet BDNF (PLT-BDNF) assays were performed.

#### 4.3.3. ELISA Assays for PPP-BDNF and PLT-BDNF Measurements

On the day of the PPP-BDNF and PLT-BDNF assays, whole-platelet pellets were removed from the −80 °C freezer, immediately placed on ice, thawed and homogenized to obtain the platelets’ soluble fractions. Briefly, the same volume of ice-cold lysis Tris-HCl buffer, pH 7.9, containing protease inhibitors (1:500, *v*:*v*), was added to resuspend and dilute each platelet pellet, homogenized by means of an immersion ultrasonic mechanical device (Vibra-Cell, Sonics, Newtown, CT, USA) and centrifuged as previously described [45,84]. The resulting supernatant, the platelet soluble fraction from lysate, was used for the PLT-BDNF assay. PPP-BDNF was instead directly apprised in thawed PPP samples. Before the test, the lyophilized BDNF standard was diluted in the sample diluent buffer in order to reach a final concentration of 1 ng/mL (1000 pg/mL). This first solution was then sequentially diluted (1:2, *v*:*v*) in the sample diluent buffer in order to obtain 7 calibration solutions, resulting in BDNF concentrations ranging from 7.8 to 500 pg/mL. The aliquots of defrosted PPP and platelet soluble fractions were also diluted in the sample buffer, carefully following the kit guidelines. Subsequently, mature BDNF standard concentrations, quality-control samples, properly diluted PPP aliquots or platelet soluble fractions and “blanks” (containing only the sample buffer) were added to all microplate wells. The plate was then covered with an appropriate plate sealer film and incubated in a thermos-plate agitator at low shaking speed (about 200 rpm) for 45 min at RT. After the incubation, the procedure featured 5 washing steps. Then, a biotinylated anti-mature BDNF antibody was added to each well, and the plate underwent an additional incubation on the thermo-agitator. Subsequently, after a supplementary washing phase, the streptavidin–horseradish peroxidase (HRP) revelation complex was added to all the wells, and then the plate was incubated again at a very low shaking speed and RT. To detect the immunocomplex formation, tetramethylbenzidine (TMB), an HRP substrate, was added to each well; this reaction was stopped by adding a strong acidic solution. This latter step converts the blue color, due to the HRP reaction’s deprotonated product, into a yellow color, due to the product’s protonated form, yielding an intensity directly proportional to BDNF concentrations. Microwell absorbance was then measured by the spectrophotometric plate reader preset at λ = 450 nm. For calculating BDNF values, the blank absorbance (Abs-450 BDNF = 0 pg/mL) was subtracted from all standard, control and sample absorbances. The BDNF values were then interpolated from a 4-PL nonlinear regression calibration curve and multiplied by the dilution factor, permitting us to obtain the final BDNF amounts in the PPP and platelet lysate fractions as ng/mL. Then, the PLT-BDNF values reported as ng/mL were normalized for the total protein amount (mg/mL) measured in each final platelet lysate fraction and converted into ng/mg proteins. The total protein amount was measured by the Bradford method [102], using γ-globulins as the standard. This correction was performed to possibly restrain the impact of the interindividual variability of platelet number on PLT-BDNF as well as to contain the varying loss of proteins during platelet homogenization and fractionation. To make possible the pairwise comparison between the plasma and platelet neurotrophin levels within the same group using the same unit of measurement, PLT-BDNF values were also analyzed as ng/mL using a correction factor obtained on the basis of the total protein amount obtained in each sample and an estimated mean value of protein content in PRP.

### 4.4. Data Analyses

All data are presented as the mean ± SD and the mean rank. All biochemical assays were conducted at least in duplicate. For BDNF calculations, the concentrations in PPP or platelet soluble fractions were interpolated as follows: the STD concentrations of BDNF, reported in the corresponding unit of measure (pg/mL), were log-transformed, and a semi-log calibration curve (*y* vs. *logx*) was built by means of a 4-PL nonlinear regression equation, as mentioned afore. After interpolating the analyte concentration in unknowns, resulting log values were then transformed again into nonlogarithmic measures (*x* = 10*^x^*). All calibration curves for biochemical dosages were carried out using the GraphPad Prism software (GraphPad, Version 8.0, San Diego, CA, USA). Due to the limited number of subjects recruited in this study and considering that normality tests and variance homoscedasticity were not respected in our sample, we applied nonparametric analyses for elaborating data. A Kruskal–Wallis one-way analysis of variance was used in order to compare continuous sociodemographic variables, psychometric instrument scores and biochemical parameter concentrations among the three groups, followed by the Dunn test for post hoc comparisons. For comparisons of continuous nonparametric variables between the two groups of BD patients, the Mann–Whitney U test was used for unpaired data, while the Wilcoxon test was employed for paired data. Chi-square tests were performed in order to compare categorical sociodemographic variables among groups. We also carried out Spearman’s correlations to obtain the R_S_ coefficient in order to evaluate the relationships between the PPP- and PLT-BDNF components as well as those between the two biochemical variables and the psychometric instrument scores. All statistical analyses were performed using SPSS version 28 (IBM Corp. 2021, Chicago, IL, USA). Due to the naturalistic and cross-sectional design of the study, for all the statistical analyses carried out, the statistical threshold was preset at *p* ≤ 0.05, whereas *p* values 0.05 < *p* ≤ 0.1 were considered as showing a trend towards significance.

## 5. Conclusions

This study, albeit preliminary, reveals that peripheral BDNF, precisely PLT-BDNF, can be related to mood symptoms rather than post-traumatic stress ones. However, due to the high heterogeneity of BD, as well as the influence on BDNF results with respect to the type and number of traumas experienced and the distance from the traumatic event in patients with post-traumatic symptoms, our findings pave the way toward additional assessments of peripheral BDNF in more homogeneous groups of PTSD patients. Because of their originality with respect to the evaluation of bipolar patients with PTSD or depressive symptoms, our results can be also considered forerunners for a deeper characterization of the circulating components of BDNF in even more defined subgroups of BD patients with specific clinical conditions, as well as in healthy subjects [46]. Indeed, present findings suggest the possible variance of the BDNF protein in the different bloodstream compartments under both physiological and pathological conditions [46,103]. Finally, BD patients with PTSD or depression and healthy controls should be investigated in association with other parameters, such as HPA activity parameters, inflammatory markers, cytokine levels, immune–hematological indices or others, with the aim of searching for patterns of variation, rather than single biochemical correlates, in larger cohorts of patients.

## Figures and Tables

**Table 1:** Comparison of psychometric instrument scores and BDNF levels among groups.

	PTSD (n = 20)	DEP (n = 20)	CTL (n = 24)			
IES-R				***Z***	***p***	**post hoc**
*Intrusion*	2.39 ± 0.90, 29.63	0.51 ± 0.36, 10.85	-	−5.156	<0.001	PTSD>DEP
*Avoidance*	2.14 ± 0.69, 29.61	0.53 ± 0.40, 10.88	-	−5.135	<0.001	PTSD>DEP
*Hyperarousal*	2.37 ± 0.75, 29.42	0.54 ± 0.37, 11.05	-	−5.047	<0.001	PTSD>DEP
*Total score*	50.42 ± 11.18, 30.00	11.55 ± 6.99, 10.50	-	−5.344	<0.001	PTSD>DEP
HAM-D			-	***Z***	***p***	**post hoc**
*Total score*	8.40 ± 2.41, 10.50	18.40 ± 3.55, 30.50	-	−5.433	<0.001	DEP>PTSD
YMRS			-	***Z***	***p***	**post hoc**
*Total score*	6.60 ± 2.70, 20.93	6.25 ± 3.73, 20.08	-	−0.232	0.736	-
BDNF				***H***	***p***	**post hoc**
PPP-BDNF, ng/mL	3.37 ± 1.70, 33.40	3.98 ± 4.67, 27.05	4.93 ± 5.49, 36.29	2.76	0.252	-
PLT-BDNF, ng/mg prot.	4.30 ± 3.27, 33.70	2.79 ± 1.54, 22.90	4.42 ± 2.40, 39.50	8.79	0.012	DEP<CTL

Data are presented as mean ± SD and mean rank referred to Mann–Whitney (**Z**) or Kruskal–Wallis (**H**) tests. **Post hoc** is referred to Dunn test after Kruskal–Wallis analyses. **IES-R**: the Impact of Event Scale-Revised; **HAM-D**: the Hamilton Depression Rating scale; **YMRS**: Young Mania Rating Scale; **PPP-BDNF**: platelet-poor BDNF; **PLT-BDNF**: intraplatelet BDNF.

## Data Availability

The data presented in this study are available on request from the corresponding author due to ethical reasons.
